# Supplementary figures and images for: Evaluation of Brace Treatment Using the Soft Brace Spinaposture: A Four-Years Follow-Up
Source: J Clin Med. 2022 Jan 5;11(1):264. doi: 10.3390/jcm11010264 (PMC8745903; doi:10.3390/jcm11010264)

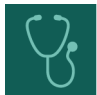

Supplement S1 Development in Cobb' angle for the 15 patients with AIS 1

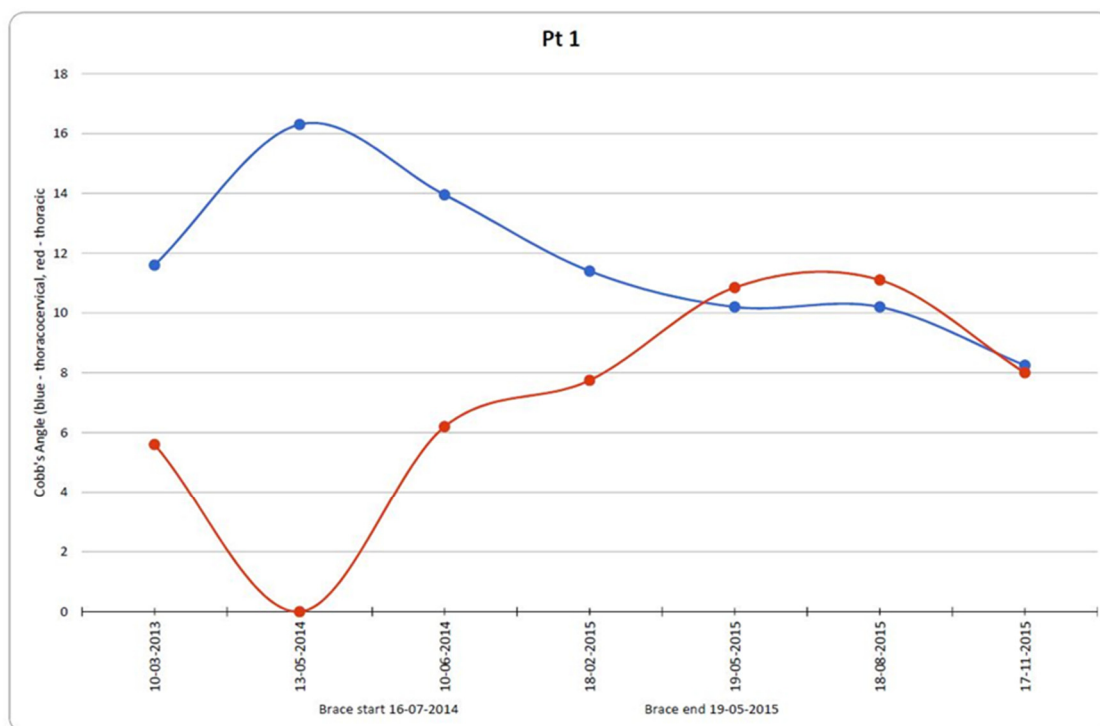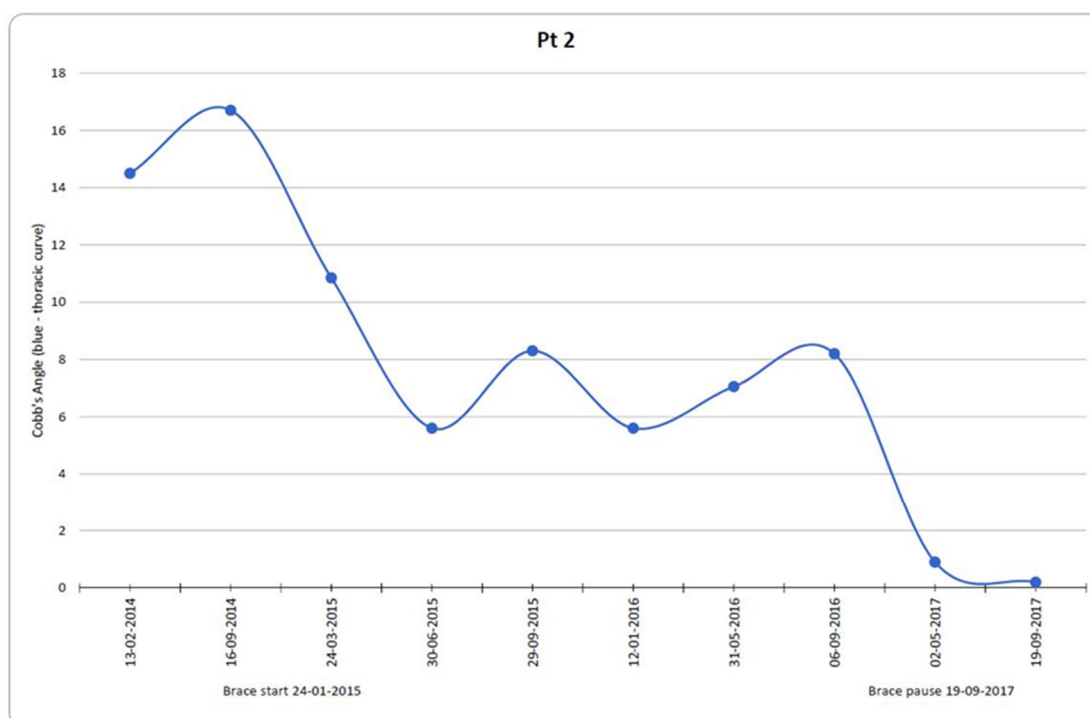

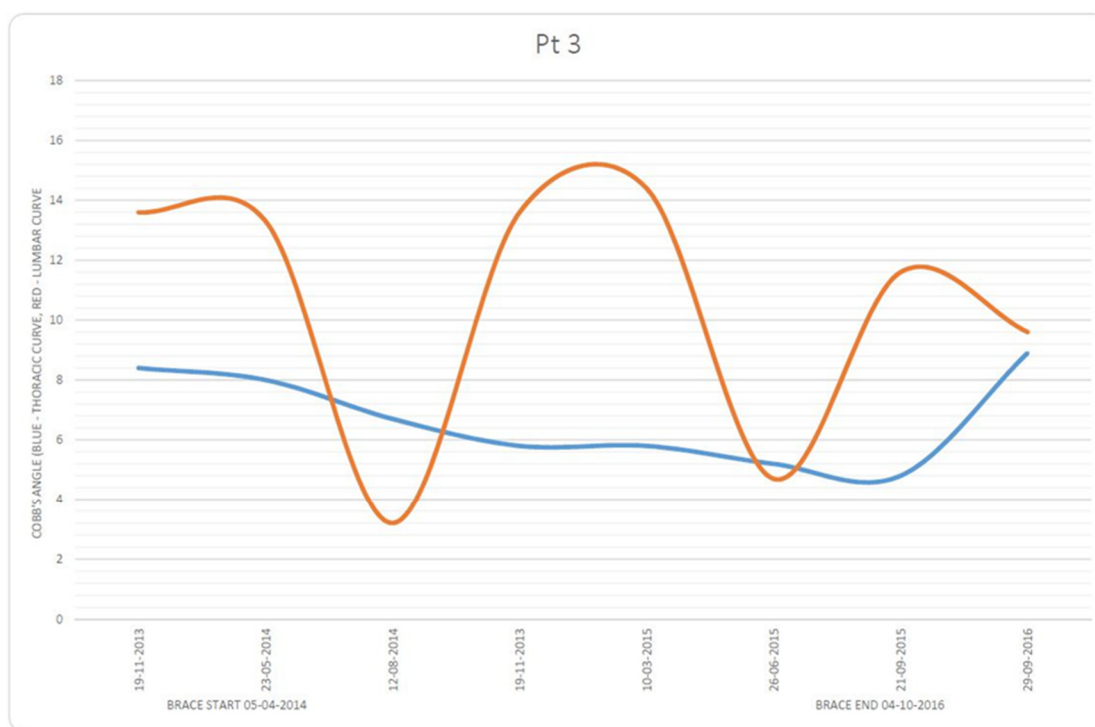

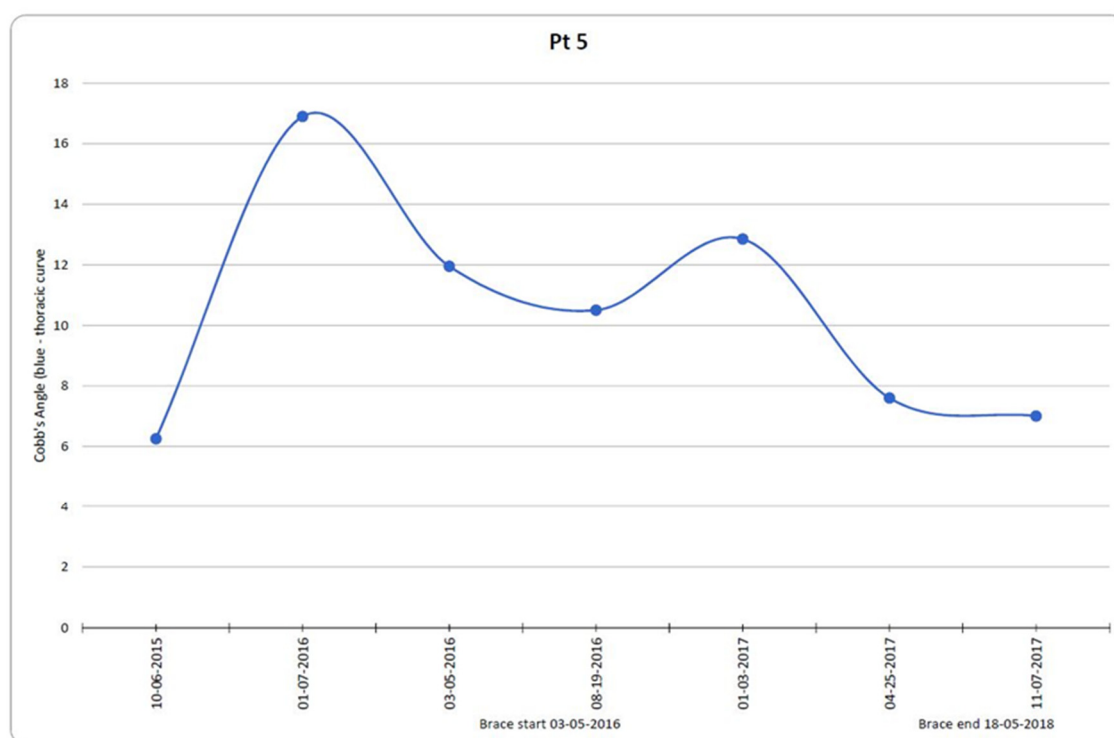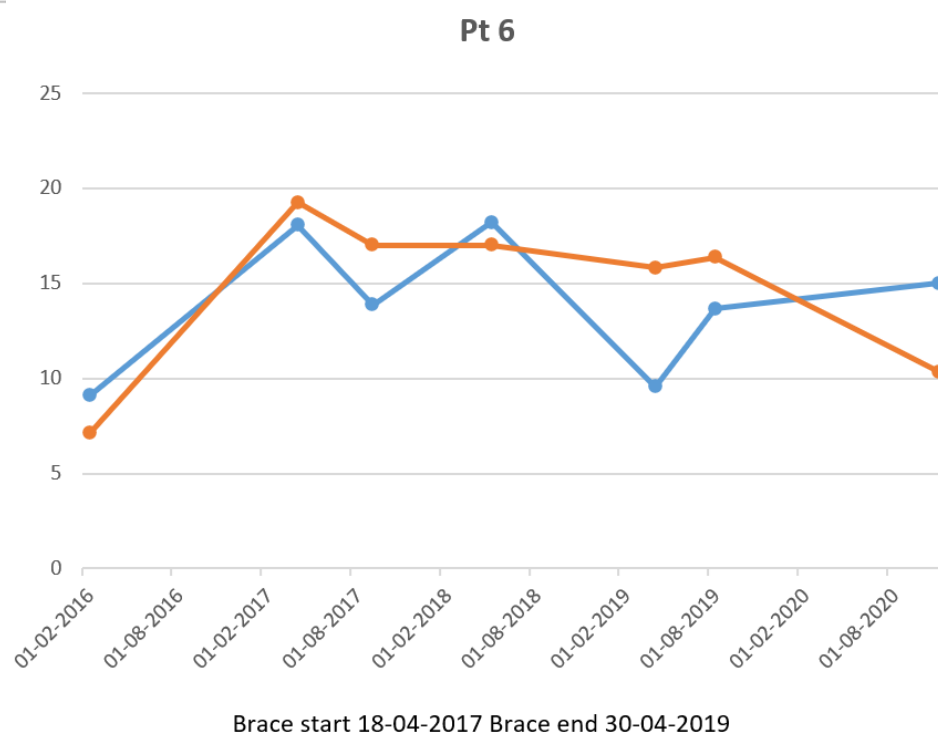

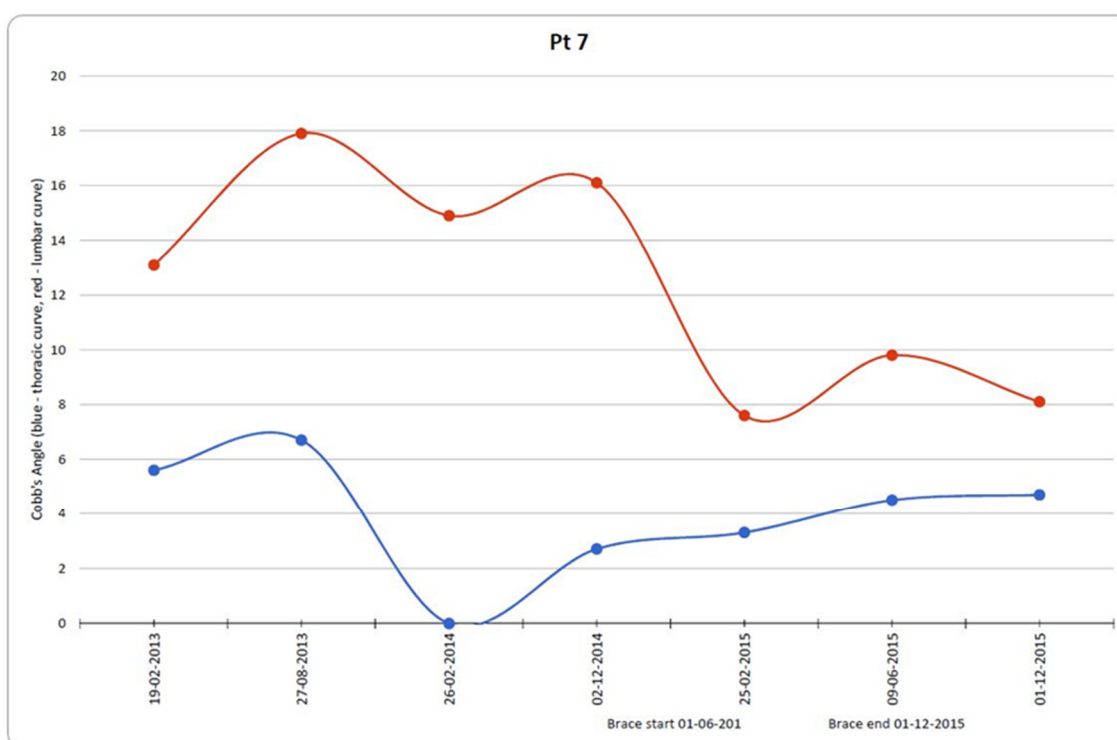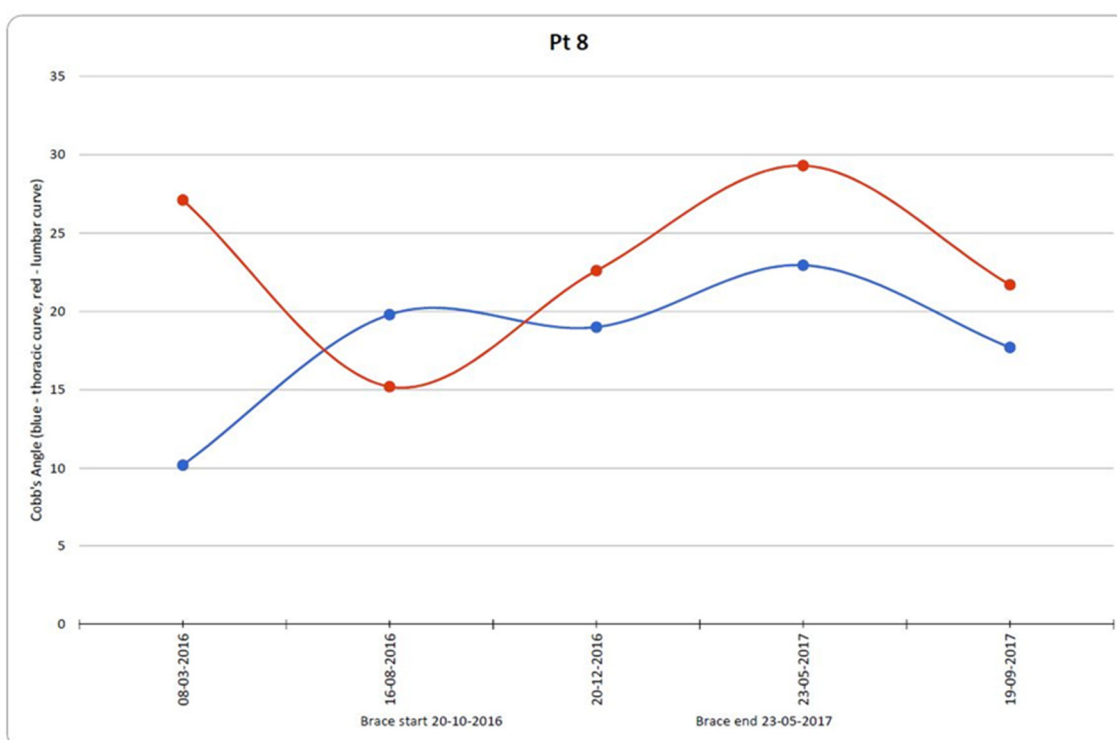

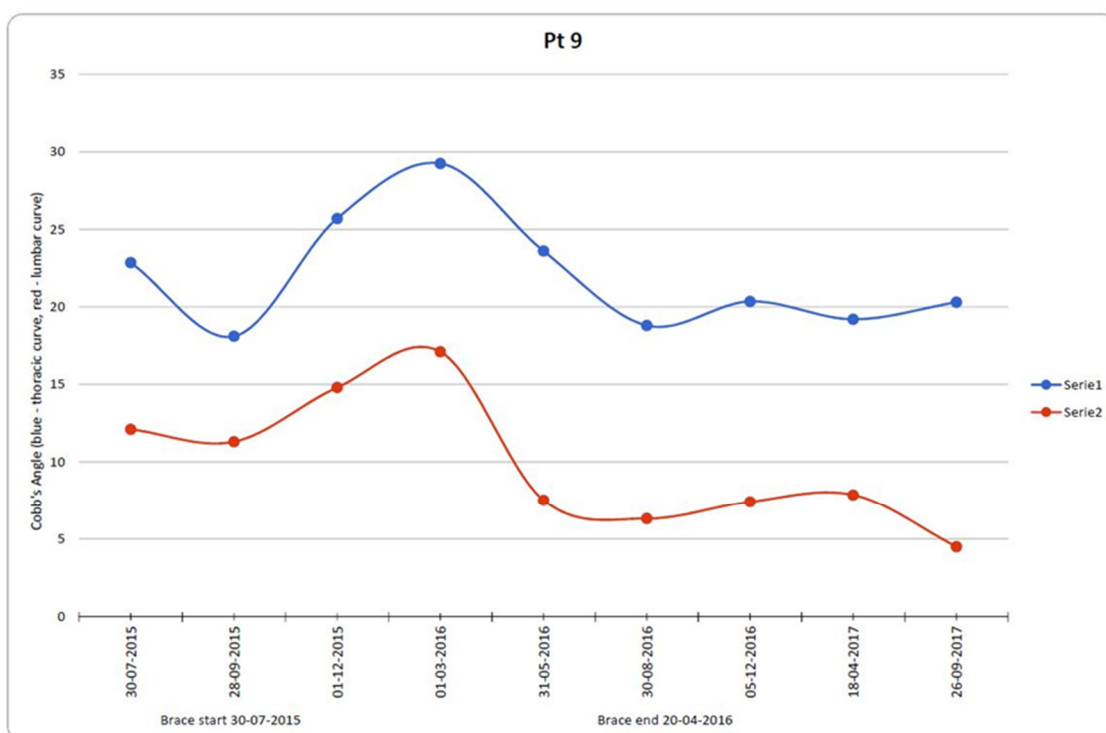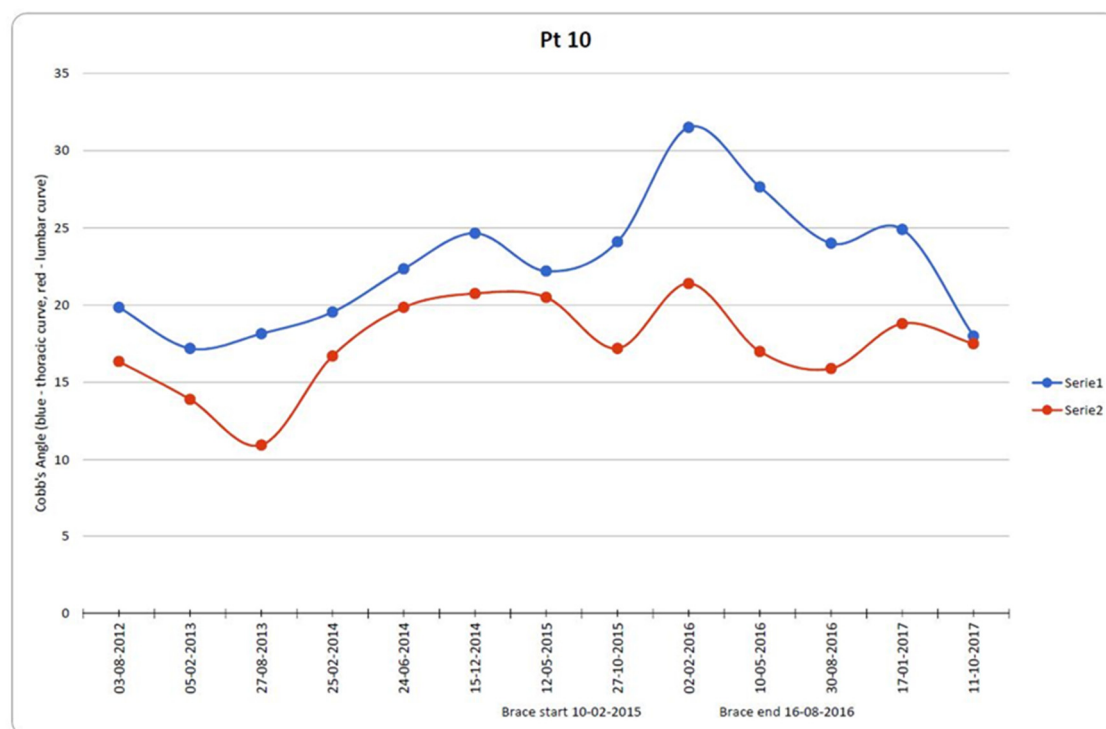

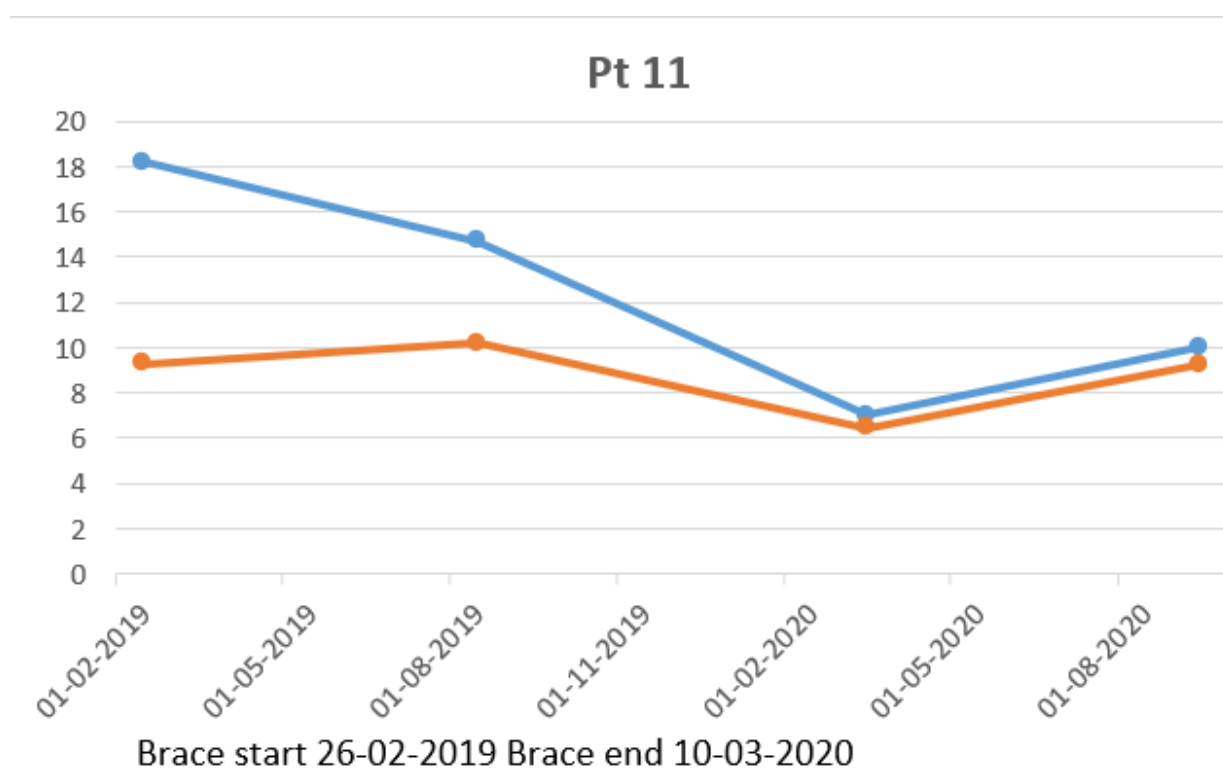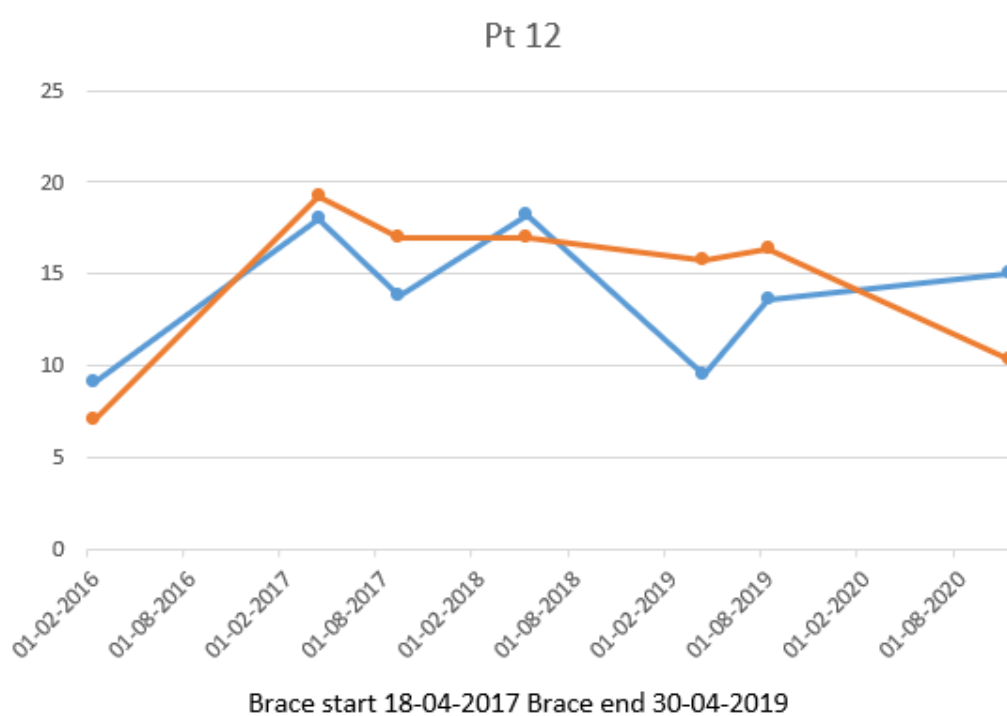

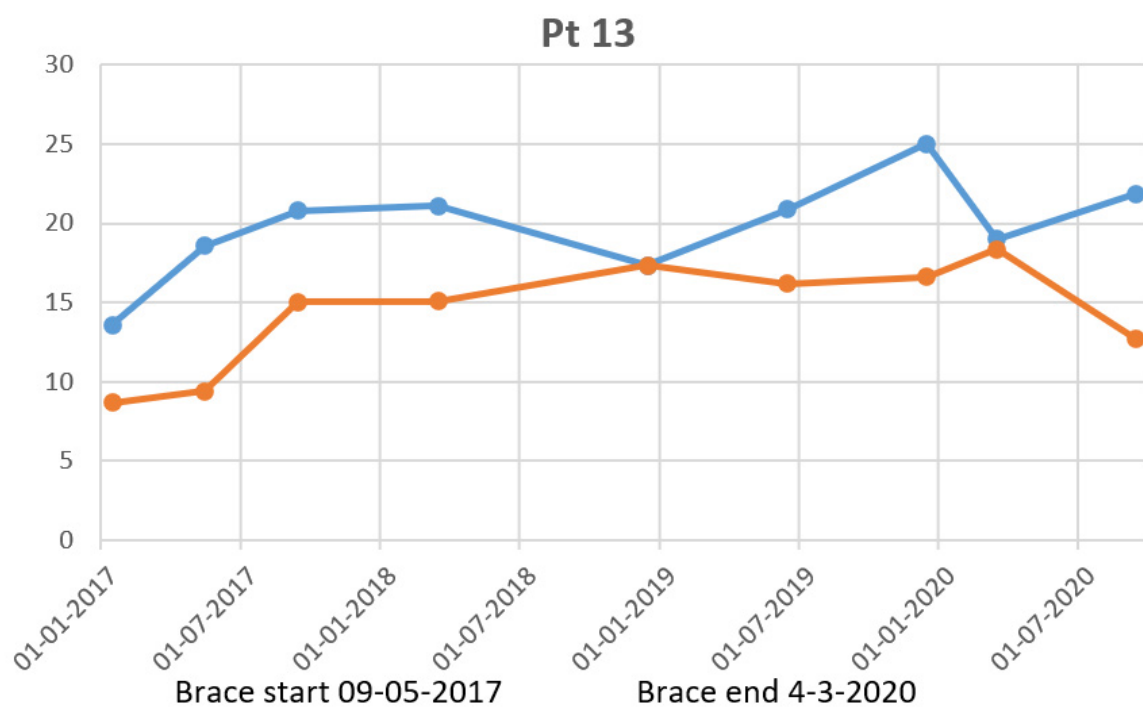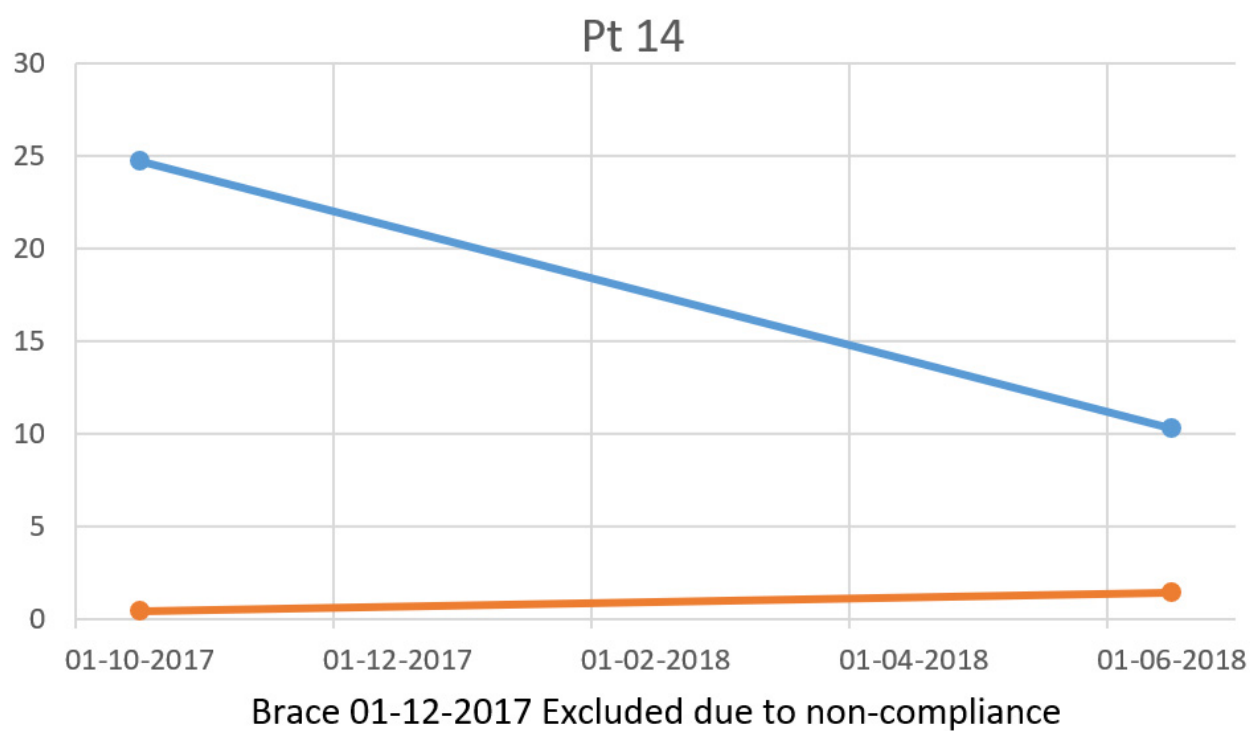

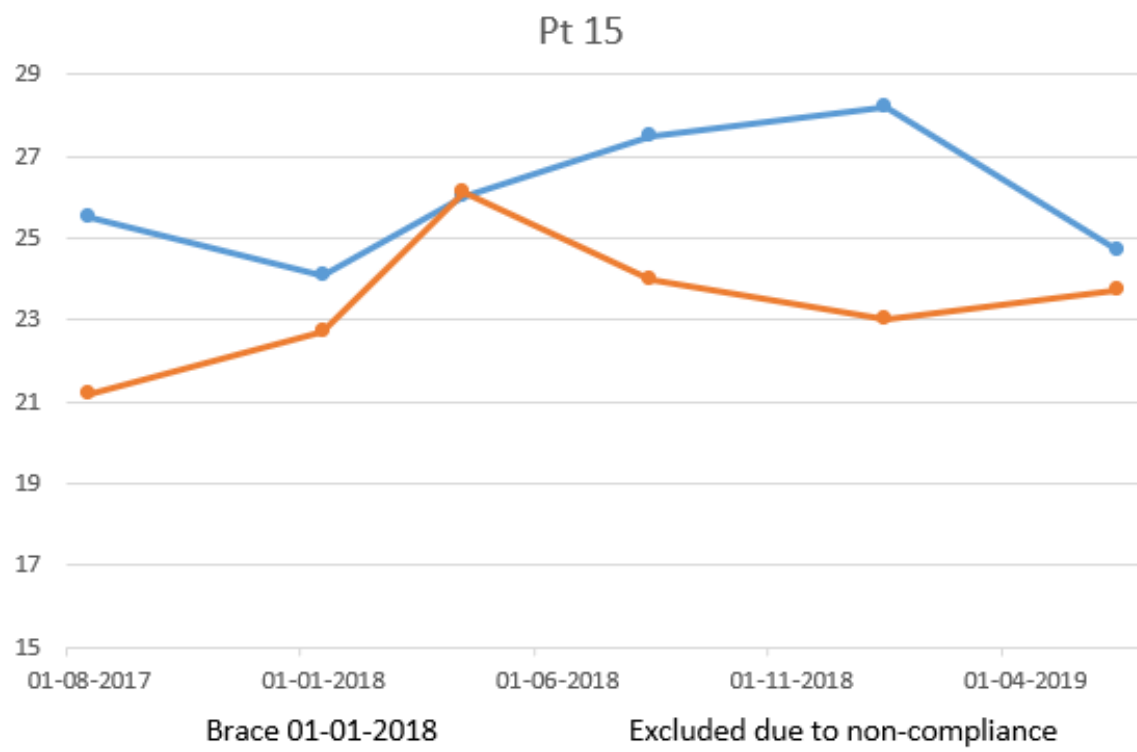

Supplement: Supplementary file 1 [file jcm-11-00264-s001.zip › jcm-1507568-SI.pdf]
